# How do study participants want to be informed about study results: Findings from a malaria trial in Cambodia, Ethiopia, Pakistan, and Indonesia

**DOI:** 10.1017/cts.2025.56

**Published:** 2025-03-27

**Authors:** Samuel Alemu Bamboro, Fareeha Abdul Jabbar, Mary Bagita-Vangana, Nurfadhilah Hasibuan, Tamiru Shibiru Degaga, Najia Ghanchi, Mohammad Asim Beg, Rupam Tripura, Ayodhia Pasaribu Pitaloka, Tedla Teferi Tego, Widya Safitri, Yulita, Sarah Cassidy-Seyoum, Muthoni Mwaura, Hellen Mnjala, Grant Lee, Lek Dysoley, Lorenz von Seidlein, Ric N. Price, Holger W. Unger, Bipin Adhikari, Kamala Thriemer

**Affiliations:** 1 College of Medicine & Health Sciences, Arba Minch University, Arba Minch, Ethiopia; 2 Department of Pathology and Laboratory Medicine, Aga Khan University, Karachi, Pakistan; 3 University of Melbourne, Faculty of Medicine, Dentistry & Health Sciences, Melbourne, Australia; 4 Port Moresby General Hospital, Obstetrics & Gynaecology Division, Port Mosby, Papua New Guinea; 5 Yayasan Penguatan Kesehatan Masyarakat Tridarma (YPKMT) / Tridarma Healthcare Empowerment Foundation (THEMP), Medan, North Sumatra, Indonesia; 6 Global and Tropical Health Division, Menzies School of Health Research and Charles Darwin University, Darwin, Australia; 7 Mahidol-Oxford Tropical Medicine Research Unit, Faculty of Tropical Medicine, Mahidol University, Bangkok, Thailand; 8 Centre for Tropical Medicine and Global Health, Nuffield Department of Clinical Medicine, University of Oxford, Oxford, UK; 9 Department of Pediatrics, Medical Faculty, Universitas Sumatera Utara, Medan, Indonesia; 10 Arba Minch General Hospital, Arba Minch, Ethiopia; 11 Department of Health Ethics and Society, Care and Public Health Research Institute (CAPHRI), Maastricht University, Maastricht, The Netherlands; 12 National Center for Parasitology, Entomology and Malaria Control, Phnom Penh, Cambodia; 13 National Institute of Public Health, School of Public Health, Phnom Penh, Cambodia; 14 Department of Obstetrics and Gynaecology, Royal Darwin Hospital, Darwin, Northern Territory, Australia; 15 Department of Clinical Sciences, Liverpool School of Tropical Medicine, Liverpool, UK; 16 Department of Infectious Diseases, University of Melbourne, Doherty Institute, Melbourne, Australia

**Keywords:** Results dissemination, research participants, clinical trial, malaria, community engagement

## Abstract

**Background:**

Researchers acknowledge the need to share study results with the patients and their communities, but this is not done consistently due to a plethora of barriers, including a paucity of data to guide best practice approaches in different populations.

**Methods:**

This study was nested within a large multi-center randomized controlled trial of antimalaria treatment. Data on dissemination preferences were collected at the third-month follow-up visit using a short questionnaire. Data were analyzed using descriptive statistics and subsequently fed into an iterative process with key stakeholders, to develop suitable strategies for result dissemination.

**Results:**

A total of 960 patients were enrolled in the trial, of whom 84.0% participated in the nested survey. A total of 601 (74.6%) participants indicated interest in receiving trial results. There was significant heterogeneity by study country, with 33.3% (58/174) of patients indicating being interested in Cambodia, 100% (334/334) in Ethiopia, 97.7% (209/214) in Pakistan, but none (0/85) in Indonesia. The preferred method of dissemination varied by site, with community meetings, favored in Ethiopia (79.0%, 264/334) and individualized communication such as a letter (27.6%, 16/58) or phone calls (37.9%, 22/58) in Cambodia. Dissemination strategies were designed with key stakeholders and based on patient preferences but required adaptation to accommodate local logistical challenges.

**Conclusion:**

The varying preferences observed across different sites underscore that a one-size-fits-all approach is inadequate. Strategies can be tailored to patient preference but require adaptation to accommodate logistical challenges.

## Introduction

Community engagement is increasingly recognized as an integral element of ethical global health research [1]. Engaging community members in health research occurs on a spectrum, and their involvement as early as possible to set priorities for research and co-design relevant research is critical [1,2]. However, involvement of community members is often deferred until the start of the recruitment processes, in line with the instrumental utility of undertaking community engagement to commence the study and ensure patient participation [3,4]. Continued community engagement after the completion of research studies, particularly dissemination of aggregated non-individualized study results to research participants, is an ethical obligation [3,5] and regulatory requirement [6,7]. Some research funding bodies now recognize the importance of these activities and require integration of result dissemination activities into research proposals [8].

Disseminating trial findings can improve health literacy and decision-making among participants, improve general understanding of research, and encourage participation in future research [9,10]. Furthermore, increased transparency and trust in medical research can lead to better satisfaction among participants [11–13]. While most researchers acknowledge the need to share aggregated study results with the patients and their communities, this is not practiced consistently [14,15].

A survey among malaria researchers showed that although more than 80% appreciated the importance of sharing results with the trial participants, only 25% accomplished this in their most recent trials [3]. Key barriers to result dissemination in those settings include difficulty locating and reaching research participants after the end of the trial [16–18], low literacy levels among study participants [3,19], limited advanced planning [3,14,15], logistical issues such as limited access to and availability of internet and phone, a lack of electricity and poor road conditions in resource-constrained settings, financial constraints [14,15], a lack of institutional guidance on how to conduct dissemination activities [3,19,20] and ethical concerns, including concerns around confidentiality within small communities [21], and fear of misinterpretations and inflicting harm [14,15].

There is a paucity of data to guide best practice for the dissemination of research findings in different study populations and settings. More commonly reported types of results sharing include written communication in letters or lay summaries [22,23], emails [23], and information placed on websites [24] or group presentations in the form of community meetings or workshops [9,25,26]. The limited literature from low- and middle-income countries comes primarily from the African continent and focuses on experiences with community meetings as the main method for result-sharing [16,25]. Most of the literature guiding the selection of methods as well as patient preferences is from high-income countries, and these methods are less likely to be relevant to participants from communities with structural barriers such as low health literacy, vulnerability, poverty, competing priorities, and access and interest to engage in learning about study findings.

Our multi-centered antimalarial trial in Cambodia, Ethiopia, Pakistan, and Indonesia was designed to compare the effectiveness, safety, cost-effectiveness, and feasibility of novel treatment options for patients with *Plasmodium vivax* malaria. As part of the trial, we conducted a nested study to assess trial participants’ preference for result dissemination after study completion to inform result-sharing strategies.

## Methods

### Study Overview

The study was conducted in two stages. In the first stage (the “survey”), data were collected on patients’ preferences for dissemination. Following the analysis of these data, the second stage involved reviewing the findings of the survey and developing suitable dissemination strategies through an iterative process among the study team.

### Study Context and Sites

**Figure 1.**
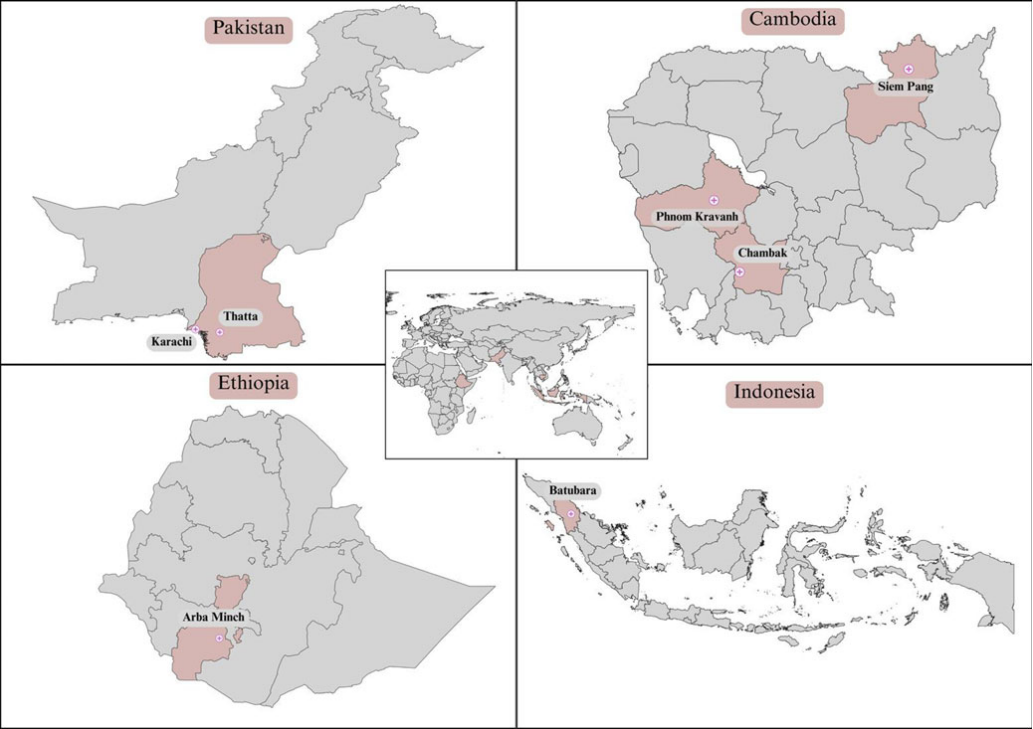
Map showing research sites in Cambodia, Ethiopia, Pakistan, and Indonesia. Generates using QGIS 3.32.3-Lima software and finalized in Canva Pro. Country Shapefiles were obtained from the Humanitarian Data Exchange (https://data.humdata.org/dataset/cod-ab-pak?, https://data.humdata.org/dataset/cod-ab-khm, https://data.humdata.org/dataset/cod-ab-eth, https://data.humdata.org/dataset/cod-ab-idn) and are licensed under a Creative Commons Attribution 4.0 (CC-BY 4.0) International licence. The world map shape file was obtained from Opendatasoft (https://public.opendatasoft.com/explore/dataset/world-administrative-boundaries/export/), and license under an Open Government Llicense v3.0.

Data for the survey were collected in the context of a multi-center clinical trial to assess the safety and effectiveness of novel approaches to the treatment *P. vivax* with tafenoquine and primaquine (NCT04411836). In brief, adult patients presenting with uncomplicated *P. vivax* malaria meeting the eligibility criteria were randomized into one of three treatment arms. After a standardized informed consent process, each patient was treated with a blood-stage treatment plus either low-dose primaquine (total dose 3.5mg/kg) unsupervised over 14 days, high-dose primaquine (total dose 7 mg/kg) unsupervised over 7 days, or a single dose of tafenoquine (300 mg). Patients were then followed weekly until day 42 and then monthly for 6 months. Patients’ recruitment occurred at seven study sites in four countries: Cambodia, Ethiopia, Pakistan, and Indonesia (Fig. 1). Training on data collection and study conduct was standardized across study sites. Background details on the study countries, malaria burden, their socio-economic structure, and literacy are summarized in Table 1.

**Table 1.** Background on study countries

	Cambodia	Ethiopia	Pakistan		Indonesia
GDP per capita (US$)*	1,759.6	1,027.6	1,588.9		4,788.0
Life expectancy at birth in years (2021)*	70	65	66		68
Literacy rate, adults total (% of people ages 15 and above)*	84% (2022)	52% (2017)	58% (2019)		96% (2020)
Mobile phone subscription per 100 people (2022)*	116	56	82		115
Fixed broadband subscriptions per 100 people (2022)*	3.04	0.46	1.33		4.88
Social Media use in percent of population (2024)^ⱡ^	68.4%	5.5%	29.5%		49.9%
Occupation and livelihood at study sites	Primarily agriculture. Community members are busy during rainy season planting rice and vegetables and may find difficult to travel during the season because of road conditions.	Mainly government, private, NGOs or self-employed. A smaller number of people in agriculture.	In urban Karachi, most people are engaged in government, private sector jobs, NGOs, or are self-employed, with a smaller number involved in agriculture.	In rural Thatta, many community members are engaged in farming and related activities	Agriculture and fishery are primary occupations. A smaller number of people work as government employee or self-employed
Malaria disease burden at study sites	Malaria transmission is heterogeneous. In 2022 there was an outbreak of falciparum malaria in Kravanh [51]. In between October-December 2023, there were a total of 276 cases (1% *P. falciparum* and mixed cases, and 94% *P. vivax* cases), a 70% decrease compared to the same time period in 2022 [52t1fn1"]. Almost half of the malaria cases between January to July 2024 (of total malaria in Cambodia) were reported from Stung Treng that hosts Siem Pang district [53]. Siem Pang had the highest malaria cases (n = 70) between January to July 2024, and almost all of them were vivax malaria. Most of these vivax malaria cases were found among soldiers, migrants, and ethnic minorities. Phnom Sruoch that host Chambak health center had high prevalence of vivax malaria in 2022, but the prevalence since then has declined with only three cases of malaria between January to July 2024 in an entire district.	Malaria occurs year-round, mainly following the rainy seasons. The peak seasons for malaria transmission in the country is from September–December. Over 80% of the malaria burden in Ethiopia is among adults and children who are at least five years of age. The The Southern Nations, Nationalities, and Peoples’ Region (SNNPR) is categorized as moderate to high disease burden with API between >10–50 for areas categorized as moderate and >50 for those categorized as high [54]. There were 415,197 confirmed malaria cases reported from SNNPR in 2022, and 126,029 (30%) were attributed to *P. vivax* [55]	Malaria occurs year-round, with higher transmission rates following the rainy seasons. In urban Karachi, malaria prevalence is relatively low (5 cases per 1000 population yearly) due to better healthcare infrastructure and urban living conditions. In rural Thatta, malaria remains a significant health issue, with higher prevalence rates (25 cases / 1000 population) attributed to agricultural practices and limited access to healthcare facilities [56].		Malaria occurs year-round with higher transmission during rainy season. Batubara is malaria moderate endemicity with 1.49 API, and one of the regencies that contributed to highest malaria cases in North Sumatera province. Ratio between *P. vivax and P. falciparum* is 70: 30 (provincial malaria data).

* based on https://data.worldbank.org/indicator.

ⱡ from https://datareportal.com/reports/digital-2024.

#### Cambodia

In Cambodia, patients were recruited at three sites: Phnom Kravanh Referral Hospital and Siem Pang and Chambak health centers. Phnom Kravanh referral hospital has a catchment population of more than 50,000 and is located in Pursat province, in western Cambodia, with the population concentrated around the Pursat River, and the western parts of the Cardamom mountains. Most communities in this area live in the forest fringe or nearby forested mountains. Although communities have access to health centers and larger health facilities, the distances between communities and these health centers can be far. Siem Pang Health Center has a catchment population of 25,000 and is located within Stung Treng province in north-eastern Cambodia, along the Tonle Kong River, surrounded by forest reserves, and national parks with abundant forest fringes. A high proportion of ethnic minorities live in Siem Pang, with difficult access to health centers, hampered by road conditions, terrain, and long distances. Chambak Health Center has a catchment population of 4,000 and is located within Phnom Sruoch, a district located within Kampong Speu province in central Cambodia. The district covers the largest part of the Kirirom National Park located in the eastern part of the Cardamom mountains. Most settlements are thus close to the forest or forest fringe. Although Chambak health center is relatively accessible, access to larger health facilities, especially among populations living in far flung communities near the forest is limited. The majority of the study team at the Cambodia sites was from within the communities where patients were recruited. In addition, international staff was present at the study sites.

#### Ethiopia

The study site in Ethiopia was located at the research facility at the Arba Minch General Hospital, Arba Minch town, in the Gamo Zone of the South Ethiopia Region. Arba Minch district is located 505 km south of Ethiopia’s capital, Addis Ababa, with a population of 320,000. The urban center has a general hospital, a primary hospital, and two health centers. In addition, there are a range of private facilities, at which approximately 20% of malaria patients seek care [27]. The study hospital receives patients from two health facilities located in Arba Minch town, Woze Health Center, and Dil Fana Primary Hospital. For urban patients, all health facilities are within walking distance or accessible by taxi or private car. The entire study team at the Ethiopian site was from within the communities where patients are recruited from.

#### Pakistan

In Pakistan, the study recruited patients at two sites: an urban site in Karachi and a more rural setting in Thatta. The Karachi site is at Khidmat-e-Alam Medical center, Nazimabad, which is located in the densely populated central district of Karachi with an estimated population of nearly 3 million. This small health care facility serves a densely populated urban area, where residents face challenges accessing health services due to overburdened facilities. The site in Thatta was at the Thatta Civil Hospital, in Sindh province with an estimated catchment population of 979,817. This rural area is characterized by scattered housing and agricultural lands, with limited access to and longer travel distances for medical care. Some members of the study team at the Pakistan site were from within the communities where patients are recruited from, other team members commuted daily to the study site from Karachi.

#### Indonesia

The Indonesian study site was located at the primary health care center in Batubara in Batubara Regency in North Sumatera province. Batubara Regency is located at the eastern shoreline of North Sumatera, with Limapuluh as its administrative seat. It covers an area of 887 km [2] with a population of 430,533. The health center serves a catchment population of nearly 34,000 people and is the primary public health facility. Approximately 45% of the population seek treatment at private facilities (personal communication head of district health office Batubara). The study team was external to the communities where patients were recruited from and worked there for the duration of the study.

### Data Collection

At the scheduled follow-up visit three months after treatment, a short survey questionnaire was used to assess patients’ preferences for study result dissemination. The questionnaire was designed based on earlier work exploring current practice of result dissemination among malaria researchers [3] and discussion among the site investigators. Patients were asked four close-ended questions by the study nurses on the following topics: i) interest in knowing the results of the trial in which they were participating (primary outcome), and if so ii) their motivation for this, iii) their preference for methods used for sharing results and iv) suitable content for the dissemination (multiple options could be selected). Following translation and pre-testing of the questionnaire among the investigators and their teams, minor adjustments were made, mostly to reduce ambiguity (Text S1). Demographic characteristics from study participants were collected as part of the data collection for the trial at enrollment into the study.

### Data Analysis

Basic descriptive statistics were used for each question, and analysis was conducted overall and per country. A multiple linear regression analysis was used to identify predictors for the primary outcome. All statistical analyses were performed using Stata version v17.0 (StataCorp, US).

### Development of Dissemination Strategies

Strategies for result dissemination were developed for each study location separately based on the results on patient preferences through an iterative process between the respective site team and the trial coordinating team. This iterative process included a virtual presentation of the country-specific findings to the site study team, followed by a discussion on how results could be translated into a strategy considering the feasibility of the preferences. Based on suggestions and agreements in the virtual meeting, a written summary strategy was drafted and shared with study teams for feedback and further discussion via email. Where necessary additional virtual meetings were conducted until agreement was reached among all team members.

### Ethics

The study was approved by the Human Research Ethics Committee of Northern Territory Health and Menzies School of Health Research (#20-3694) and country-specific institutional, national, and regulatory authorities (Table S1). All patients provided informed consent.

## Results

### Study Population

A total of 960 adult patients were enrolled in the clinical trial, 220 in Cambodia, 350 in Ethiopia, 240 in Pakistan, and 150 in Indonesia. Data on dissemination preferences was obtained from 806 (84.0%) of the trial participants, of whom 174 (79.1%) were recruited in Cambodia, 334 (95.4%) in Ethiopia, 214 (89.2%) in Pakistan, and 84 (56.0%) in Indonesia. Non-participation in the survey only occurred when the scheduled follow-up visit at month three post treatment was missed. The demographic characteristics of patients who participated in the survey were similar to those for whom no data was collected (Table 2, Table S2).

**Table 2.** Demographic characteristics of study participants by study country

		Cambodia *N* = 174	Ethiopia *N* = 334	Pakistan *N* = 214	Indonesia *N* = 84	Total *N* = 806
Sex	Male	155 (89.1%)	188 (56.3%)	155 (72.4%)	53 (63.1%)	551 (68.4%)
	Female	19 (10.9%)	146 (43.7%)	59 (27.6%)	31 (36.9%)	255 (31.6%)
Age in years*	16-<18	0 (0%)	0 (0%)	0 (0%)	15 (17.9%)	17 (2.3%)
	18–30	113 (64.9%)	256 (77.0%)	100 (46.7%)	31 (36.9%)	462 (62.6%)
	31–60	61 (35.1%)	76 (22.8%)	106 (49.5%)	37 (44.1%)	251 (34.0%)
	>60	0 (0%)	1 (0.3%)	8 (3.7%)	1 (1.2%)	8 (1.1%)

* The study site in Indonesia allowed recruitment of patients≥16years, while the other study sites only recruited patients≥18years.

### Interest in Learning About Study Results

Overall, 74.6% (601/806) of participants indicated they were interested in learning about the study result. This was different by study country with 33.3% (58/174) of participants in Cambodia being interested in receiving aggregated study results, compared to 100% (334/334) in Ethiopia, 97.7% (209/214) in Pakistan, and 0% (0/85) in Indonesia (p < 0.005). A total of 82.3% (210/255) female patients compared to 71.1% (391/551) male patients indicated interest in learning about study results (p = 0.001). Interest across age groups was distributed as follows: 100% (15/15) among the 16–18-year-olds, 79.8% (400/501) among those 18 to 30 years, 68.6% (192/280) among patients 30 to 60 years and 90% (9/10) among those over 60 years old (p < 0.001).

In the multivariable analysis, the differences between sites remained significant (p < 0.001) after controlling for age and sex.

### Motivation for Hearing About Study Results

In total 45.6% (274) of the 601 patients who expressed an interest in hearing about the study results indicated that the main reason was to understand the benefit of the study to the community. A further 16.3% (98) patients indicated they were interested to understand the study in an accessible and easy way, and 38.1 % (229) patients indicated that it represented an acknowledgment of their contribution to the study. Motivation for wanting to hear results differed by country, with 76.6% (160/209) of patients in Pakistan indicating acknowledgment of their contribution as the most important reason, while understanding the benefit of the study to the community was selected by 71.9% (240/334) patients in Ethiopia (Table 3).

**Table 3.** Reason for dissemination by study country

	Cambodia *N* = 58	Ethiopia *N* = 334	Pakistan *N* = 209	TOTAL *N* = 601
To understand the benefit of the study to the community	24 (41.4%)	240 (71.9%)	10 (4.8%)	274 (45.6%)
Acknowledgement of my contribution to the study	17 (29.3%)	52 (15.6%)	160 (76.6%)	229 (38.1%)
To understand the study in an accessible and easy way	17 (29.3%)	42 (12.6%)	39 (18.7%)	98 (16.3%)

### Preference for Dissemination Methods

Participant preference for methods by which study results are communicated varied by country. A large majority of patients in Pakistan (99.0%; 207/209) felt that it was important to have summaries in their own language, while this was only reported by 38.3% (128/334) in Ethiopia. Cambodian patients preferred receiving a letter (27.6%; 16/58) or a phone call from someone explaining the study results (37.9%; 22/58), while Ethiopian patients overwhelmingly preferred a community meeting at the clinic or health center (79.0%; 264/334). In Pakistan, a high preference was indicated for having the results published on a website (60.8%; 127/209), a personal phone call to explain results (76.1%: 159/209), or a community meeting (92.3%; 139/209) (Table 4).

**Table 4.** Preference for dissemination methods by study country

	Cambodia *N* = 58	Ethiopia *N* = 334	Pakistan *N* = 209	TOTAL *N* = 601
I prefer a meeting at the Clinic/ health center together with other trial participants and someone explain the results	5 (8.6%)	264 (79.0%)	139 (92.3%)	462 (76.9%)
I prefer a summary in my language	45 (77.6%)	128 (38.3%)	207 (99.0%)	380 (63.2%)
I prefer someone to call me and explain the study result	22 (37.9%)	7 (2.1%)	159 (76.1%)	188 (31.3%)
Published on Website	0 (0%)	24 (7.2%)	127 (60.8%)	151 (25.1%)
Sent via Text/ WhatsApp/ Messenger	9 (15.5%)	20 (6.0%)	43 (20.6%)	72 (12.0%)
I prefer someone to come to my house and explain the study results in person	3 (5.17%)	19 (5.7%)	33 (15.8%)	55 (9.2%)
Via letter	16 (27.6%)	1 (0.3%)	10 (4.8%)	27 (4.5%)
Via email	0 (0%)	14 (4.2%)	2 (1.0%)	16 (2.7%)
Published on Twitter/X	0 (0%)	7 (2.1%)	5 (2.4%)	12 (2.0%)

In Cambodia and Ethiopia, most patients selected three or less options for methods of dissemination (57/58, 98.3% and 329/334, 98.5%), whereas in Pakistan 66.0% (138/209) selected more than 3 options (Fig. S1–3).

### Preference for Dissemination Content

Almost all patients in Pakistan (94.3%, 197/209) and Ethiopia (93.7%; 313/334) felt that it was important to include information about the purpose of the study in the dissemination summary. Whereas patients in Pakistan felt that it was important to include information about medical treatment (84.7%; 117/209) and scientific advances (75.6%; 158/209) based on the study’s results. Patients in Ethiopia were interested to hear about how the study was conducted (39.5%, 132/334) and the potential implications of the results to change treatment policy (30.2%; 101/334). Cambodian patients had limited interest to hear about general scientific advances (1.7%; 1/58), potential policy change (0%; 0/58), or new research based on the results (0%; 0/58). Overall patients felt it was more important to include positive findings (72.1%; 333/601) compared to negative (11.0%; 66/601) or neutral results (7.3%; 44/601) (Table 5).

**Table 5.** Preference for content of dissemination by study country

	Cambodia *N* = 58	Ethiopia *N* = 334	Pakistan *N* = 209	TOTAL *N* = 601
Purpose of the study	32 (55.2%)	313 (93.7%)	197 (94.3%)	542 (90.2%)
Good results	33 (56.9%)	214 (64.1%)	186 (89.0%)	333 (72.1%)
General scientific advances based on the study’s results	1 (1.7%)	57 (17.1%)	158 (75.6%)	216 (35.9%)
Medical treatment advances based on the study’s results	8 (13.8%)	67 (20.1%)	177 (84.7%)	152 (31.9%)
How the study was conducted	4 (6.9%)	132 (39.5%)	31 (14.8%)	176 (27.8%)
Potential policy changes based on the study’s results	0 (0%)	101 (30.2%)	39 (18.7%)	140 (23.3%)
Potential new research based on the study’s results	0 (0%)	84 (25.2%)	47 (22.5%)	131 (21.8%)
Results that are specific to me	18 (31.0%)	47 (14.1%)	61 (29.2%)	126 (21.0%)
Bad results	8 (13.8%)	29 (8.7%)	29 (13.9%)	66 (11.0%)
Neutral results	3 (5.2%)	6 (1.8%)	35 (16.8%)	44 (7.3%)

### Dissemination Strategies

Separate dissemination strategies for Cambodia, Ethiopia, and Pakistan were developed based on the survey results to inform the target audience, the method of delivery, and the content. No strategy was developed for the Indonesian site, given the lack of local interest. Although 23.2% (126/601) of participants indicated that they wanted to hear about individual results, this was not within the scope of our dissemination efforts. Draft strategies are summarized in Table 6.

**Table 6.** Draft dissemination strategies

	Cambodia	Ethiopia	Pakistan
Target population	All patients who indicated they are interested in hearing about the study results	All recruited patients	All recruited patients
Approach	Patients will be selected from the study database based on their individual identifiers and contact information will be derived from a separate patient’s information log, which was used to support patient follow up. If phone numbers are available and still functioning, patients will be contacted by senior staff members and asked if they want to hear about study results on this phone call. An option to arrange for a call at a later time point will be provided. If patients agree a short pre-written summary will be presented verbally. Participants will also be offered option to receive the written summary in addition or as an alternative to the phone conversation. This will be sent via WhatsApp/ messenger.	Patients will be contacted using the contact information collected in the patient information log and invited to a community meeting at the study center. Given the large number of patients multiple such meetings might be conducted. In line with previous similar meetings at the study site [25], the site investigator will provide a verbal summary followed by questions and answer session.	A two-pronged approach will be used: firstly, patients will be contacted using the contact information collected in the patient information log and invited to a community meeting at the study center. Given the large number of patients multiple such meetings might be conducted. Given the importance of acknowledgement of participant contribution the meetings will be framed as “thank you” meetings as opposed to focusing on information sharing. Secondly, an information package will be prepared to be shared on a webpage and patient will be informed about this in conjunction with the invite to the community meeting. The online package will include written information as well as a video. This will be after publication of study results.
Additional measures	Given the history of the site conducting community meetings to disseminate results [57], participants will also be offered an option to come to the health center for an additional meeting to discuss on the study findings.	In line with previous experience [25,57] the community meetings will be accompanied by refreshments supporting further informal conversations aimed at helping participants gain a deeper understanding of the study’s goal and potentially become community advocates for forthcoming activities including the new studies.	The investigator team opted against providing information at individual bases given the logistical difficulties and time commitments this would require. However, if participants request additional information, they will be offered a one-on-one meeting.
Content focus	Verbal summary as well as the written summary will include information on the purpose of the study, medical treatment advances based on the study’s results as well as a short summary of how the study was conducted.	The verbal communication during the meeting will include information on the purpose of the study, how the study was conducted, the implications for medical and scientific advances as well as implications for new research and policy. A focus will be given for participants to appreciate the benefits of the study to the community.	Both the verbal communication during the meeting as well as the online information will be framed to acknowledge participants participation and thank them for their contribution. Information provided will include information on the purpose of the study as well as the implications for medical and scientific advances. Online information will only be available after the study results are published.

## Discussion

Our study highlights marked heterogeneity between study countries in the interest of hearing about the results of the clinical trial that they had been enrolled in, preferred methods of dissemination, and the content to be included in dissemination activities. None of the Indonesian patients expressed an interest in hearing about the study results, while all of the Ethiopian patients indicated interest. In Ethiopia, the preferred method for dissemination was a community meeting, whereas in Cambodia patients preferred more individualized communication. In Pakistan, patients had strong preference for community meetings, individual communication as well as web-specific distribution. While most patients wanted to hear about study results in their own language and wanted to be reminded about the purpose of the study, there were large differences in the degree of interest in the impact on medical or scientific advances and the implications for policy.

Our study underscores the importance of contextualizing dissemination strategies according to social, cultural, and research literacy-related characteristics of the population rather than adopting a one-size-fits-all approach. The results also indicate the potential added benefit to conduct formative research alongside clinical trials so that community preferences can be explored, and community engagement strategies tailored to local populations [28,29]. Previous studies have demonstrated that formative research is deemed to be critical for optimal design and implementation of interventions and programs [30–33]. However, in practice, these processes are constrained by the available resources and how they can be used, which in turn affects the degree of involvement of research participants [2]. Community engagement usually occurs on a wide spectrum of activities from simply asking about preferences to incorporating community opinions at study inception through co-design [34–36]. While our survey focused primarily on exploring participants’ preferences for post-trial engagement, these engagement activities (exploring participants’ preferences) even if they are subtle, can demonstrate respect, and forge providence for future research, sustaining trust and relationships [36–38].

The ethical obligation to disseminate research findings to participants can conflict with respecting the agency and autonomy of patients, particularly if they prefer not to be informed about the trial results [39]. None of the Indonesian study participants indicated interest in hearing about the trial results and therefore no dissemination strategy was developed for this study population. While further qualitative research is required to fully understand motivations and drivers, a potential explanation for this finding based on internal team discussions might be the fact that the study team was external to their community which may have fostered a sense of difference: “us” versus “them” thus deterring community members from further engagement on results dissemination. Other generic barriers potentially include socio-economic constraints that restrict participants’ time, and opportunity costs incurred whilst engaging in results-dissemination efforts. These factors may also have contributed to the relatively large number of participants who were lost to follow up at the Indonesian site. However, it remains unclear how much of a role this played compared to other sites where patients are coming from similarly low socio-economic backgrounds.

Preferences of engagement activities can vary widely based on cultural, educational, and infrastructural factors [9,19,29,40]. For example, the strong preference for community meetings in Ethiopia and Pakistan is consistent with studies indicating that face-to-face communication is often more effective in low-resource settings where digital literacy and internet access may be limited [41,42]. The results are in line with previous practice at the study site in Ethiopia [25], potentially indicating that participants selected choices they are more familiar with. While there was a clear preference for community meetings in Ethiopia, in Pakistan a preference for individual communication was also stated, which aligned more with Cambodian patients. The preference for personalized communication in Cambodia likely reflects participants’ desire not to be engaged frequently, owing to their involvement in agricultural work coupled with barriers to reaching the health center [43,44]. Sometimes, community members may feel participation in research and engagement itself as a burden [1]. Cambodian participants have been reported to exhibit social tendencies of conformism, quietness, and hierarchical etiquette with researchers and a reluctance to “losing face” [45]. In response to these social and cultural characteristics and to bridge the differences between researchers and community members, in Siem Pang, a youth advisory group on health and research engagement was recently established [36,37].

Patients at the Pakistani study sites were the only ones indicating a high preference for digital communication, including a webpage or digital messaging services. This preference was evident in both urban and rural settings. Given that the large majority of patients were recruited in the rural site, no meaningful comparison between sites was possible (data not shown). Given the relatively low literacy rate in Pakistan coupled with digital inequality [41] these results are surprising, requiring further exploration. Digital communication allows reviewing information at convenient times for the individual and reducing additional opportunity costs for travel and in-person meetings, which might be relevant in this patient cohort.

Our findings also revealed heterogeneity in preferences about the content of the dissemination. While there was a high interest to learn more about implications for the community in Ethiopia in line with the preference for community-based delivery methods, there was a lack of interest in policy implication in Cambodia, which might be explained as a function of a research-saturated setting.

The initial design of our dissemination strategies was based on patient preferences; however, adaptations were needed to accommodate the realities of what investigator teams considered logistically and financially feasible. For example, individual phone calls to each participant were considered impractical by the study team in Pakistan. This highlights that patient preferences do not necessarily align with what investigators perceive as being feasible.

A substantial number of patients indicated they were interested in learning about individual test results arising from the trial. Although some participants may conflate the difference between sharing of aggregated and individual results, the impact for participants is likely to be similar [46]. There are however a number of considerations that are distinctly different between sharing aggregated study results versus individual test results including concerns around confidentiality, interpretation of results, and implications for further care [47]. Most of the research on returning individual test results to study participants comes from genomic research where interpretation of results remains challenging [48]. However, studies indicate that some participants prefer to receive individual results even if they have no clinical significance [49]. Our preliminary qualitative research conducted in Cambodia (unpublished data) suggests that communication on individual results during the follow up is important to patients and could affect perceptions on dissemination of overall study results and engagement in future trials.

Our study has several limitations. Firstly, the questionnaire was developed based on previous work assessing current practice of result dissemination among malaria researchers [3], but was not co-designed through formative qualitative work with patients. Therefore, the selection of choices both for methods as well as content of dissemination may not have included the entire breadth of possibilities relevant to participants. However, none of the participants used the “other” option that was designed to capture additional concepts. Second, no data on socio-economic, ethnic, or educational background was collected. This would have allowed for a more granular analysis of our findings for different populations. Third, in line with the limitations of quantitative surveys, the data does not provide explanations as to the reasons behind the preferences and more qualitative research would be needed to explore this in depth. Fourth, although training was standardized across study sites, we cannot exclude that answers to questions were not influenced by social desirability bias, or by the way study teams asked questions differently depending on their preconceived ideas. This includes potential differences in the extent of information that was provided as part of the consenting process. Fifth, patients recruited into the trial and surveyed do not necessarily reflect the overall population, and thus their preferences may not be generalizable for the larger population, this includes potential differences by gender. The majority of participants in the trial were male and this bias towards male patients was particularly strong in the Cambodian site. This reflects the epidemiology across the Greater Mekong subregion where malaria is predominantly a disease of young males with increased occupational risks (e.g. forest goers) to get infected [50]. Lastly, at the time of writing the results of the trial were not yet available, therefore, the developed dissemination strategies include only high-level guidance on content. In addition, no evaluation of the impact and feasibility of the different strategies has been conducted.

In conclusion, our study highlights the critical need for tailored dissemination strategies in global health research. The varying preferences observed across different countries underscore that a one-size-fits-all approach is inadequate. Researchers must consider patient preferences and context when planning dissemination activities.

## Supporting information

Bamboro et al. supplementary material Bamboro et al. supplementary material

## Supplementary material

The supplementary material for this article can be found at https://doi.org/10.1017/cts.2025.56.
