# Supplementary material for: How do study participants want to be informed about study results: Findings from a malaria trial in Cambodia, Ethiopia, Pakistan, and Indonesia
Source: J Clin Transl Sci. 2025 Mar 27;9(1):e83. doi: 10.1017/cts.2025.56 (PMC12086731; doi:10.1017/cts.2025.56)
Supplement: Bamboro et al. supplementary material [file S2059866125000561sup001.docx]

Contents

[Text S1: Data collection instrument for dissemination preferences 2](#_Toc196807934)

[Table S1: Overview of ethical review boards 4](#_Toc196807935)

[Table S2: Demographic characteristics of study participants not included in dissemination preference survey 6](#_Toc196807936)

[Figure S1: Selection of multiple choices for dissemination methods in Cambodia 7](#_Toc196807937)

[Figure S2: Selection of multiple choices for dissemination methods in Ethiopia 8](#_Toc196807938)

[Figure S3: Selection of multiple choices for dissemination methods in Pakistan 9](#_Toc196807939)

## Text S1: Data collection instrument for dissemination preferences

| **VISIT 10/ MONTH 3** | | |
| --- | --- | --- |
| **RESULT DISSEMINATION QUESTIONNAIRE** | | |
| 1. After the study is completed and all the data is analyzed (this could be several months after your last follow up visits) do you want to learn about the results of the trial? | |  Yes ***If yes, continue to question 2.***   No ***If no, stop here*** |
| 1. How would you like to be informed about the trial results?   ***Please select all that apply.*** | |__| I prefer a summary in my own language  |__| Via Letter  |__| Via Email  |__| Published on a website  |__| Published on Twitter  |__| Sent via Text/WhatsApp/Messenger  |__| I prefer someone calls me and explains the study results  |__| I prefer someone comes to my house and explains the study results in person  |__| I prefer a meeting at the clinic/health centre together with other trial participants and someone explains the results  |__| Other  **Please Specify:** | |
| 1. What do you feel is the ***most*** important reason for hearing the results of the trial? ***Choose one.*** | |__| acknowledgement of my contribution to the study  |__| To understand the study in an accessible and easy way  |__| To understand the benefit of the study to the community | |
| 1. What type of information should be included in the results information?   ***Please select all that apply.*** | |__| Purpose of the study  |__| How the study was conducted  |__| Medical treatment advances based on the study’s results  |__| General scientific advances based on the study’s results  |__| Potential policy changes based on the study’s results  |__| Potential new research based on the study’s results  |__| Results that are specific to me  |__| Good results of the study  |__| Bad results of the study  |__| Neutral results of the study  |__| Other  **Please Specify:** | |

## Table S1: Overview of ethical review boards

| Australia | The Human Research Ethics Committee of the Northern Territory Department of Health (HREC)  John Mathews Building (Bldg 58)  Royal Darwin Hospital Campus, Rock  PO Box: 41096, Casuarina NT 0811, Australia  Website: www.menzies.edu.au |
| --- | --- |
| United Kingdom | The Oxford Tropical Research Ethics Committee (OxTREC )  University of Oxford  Research Services, University Offices  Willington Square, Oxford OX1 2JD  Tel: +44 (0) 1865 (2) 82106  E-mail: oxtrec@admin.ox.ac.uk  Website: www.admin.ox.ac.uk/rso/ |
| Ethiopia | The National Research Ethics Review Committee (NRERC) Addis Ababa, Ethiopia  PO Box: 2490 Tel: +251 114-674-353 E-mail: most@ethionet.et Fax: +251 114-660-241 Website: www.most.gov.et |
|  | Scientific & Ethical Review Committee (SERC) Ethiopian Public Health Institute Addis Ababa, Ethiopia  PO Box: 1242/5654 E-mail: ephi@ethionet.et Tel: +251 11 2133499, +251 11 2751522 Fax: +251 11 2758634 Website: www.ephi.gov.et |
|  | The Food Medicine and Health Care Administration and Control Authority (FMHACA) Addis Ababa, Ethiopia  Tel: 251-11-552 41 22/552 41 23 E-mail: regulatory@fmaca.gov.et Fax: 251-11-552 13 92 PO Box: 5681 |
| Indonesia | Health Research Ethical Committee  Medical Faculty of Universitas Sumatera Utara/ H. Adam Malik General Hospital  Jl. Dr. Mansyur No 5 Medan, 20155 - Indonesia  Tel: +62-61-8211045; 8210555 Fax: +62-61-8216264 E-mail:  komisietikfkusu@yahoo.com |
|  | Indonesian Food and Drug Agency (BPOM)  Jl, Percetakan Negara No. 23 Jakarta Pusat 10560 Indonesia E-mail: infopom@indo.net.id; Tel: (021) 4244691, 4209221, 4263333, 4244755, 4241781, 4244819 Fax: (02) 4245139 Website: www.pom.go.id |
| Cambodia | National Ethics Committee for Health Research, Ministry of Health, Kingdom of Cambodia,  Lot #80, Samdach Penn Nouth Blvd (289), Sangkat Boeung Kok 2, Khan Tuol Kork, Phnom Penh, Cambodia. Tel: (855~12) 842 442, (855-012) 528 789, (855-012) 203 382 |
| Pakistan | Ethics Review Committee, Aga Khan University,  Stadium Road, P.O. Box 3500 Karachi 74800, Pakistan. Telephone: +92 21 3493 0051 Ext: 2447/4988. Email: erc.pakistan@aku.edu |
|  | National Institutes of Health, Health Research Institute, National Bioethics Committee (NBC),  Shahrah-e-Jamhuriat, Off Constitution Avenue, Sector G-5/2, Islamabad  www.nbcpakistan.org.pk,e-mail: nbcpakistan@nih.org.pk Tel: 92-51-9224325, 9216793, Fax 9216774 |
|  | Drug Regulatory Authority of Pakistan, Pharmacy Services Division Prime Minister's National Health Complex, Park Road, Chak Shahzad, Islamabad, Pakistan, Telephone: +92 51 9107316, Email: ceo@dra.gov.pk |

## Table S2: Demographic characteristics of study participants not included in dissemination preference survey

|  | | Cambodia  N=220 | Ethiopia  N=350 | Pakistan  N=240 | Indonesia  N=150 | TOTAL  N=960 |
| --- | --- | --- | --- | --- | --- | --- |
| Number of participants not participating in the dissemination preference survey | | 46 (2.9%) | 16 (4.6%) | 26 (10.8%) | 66 (44.0%) | 154 (16.0%) |
| Sex | Male | 43 (93.5%) | 9 (56.3%) | 22 (84.6%) | 47 (71.2%) | 121 (78.6%) |
|  | Female | 3 (6.5%) | 7 (43.8%) | 4 (15.4%) | 19 (28.8%) | 33 (21.4%) |
| Age in years | 16-<18 | 0 (0%) | 0 (0%) | 0(0%) | 8 (12.1%) | 8 (5.2%) |
|  | 18-30 | 32 (69.6%) | 14 (87.5%) | 18 (69.2%) | 32 (48.5%) | 96 (62.3%) |
|  | 31-60 | 14 (30.4%) | 2 (12.5%) | 7 (26.9%) | 25 (37.9%) | 48 (31.2%) |
|  | >60 | 0 (0%) | 0 (0%) | 1 (3.9%) | 1 (1.5%) | 2 (1.3%) |

## Figure S1: Selection of multiple choices for dissemination methods in Cambodia

## Figure S2: Selection of multiple choices for dissemination methods in Ethiopia

## Figure S3: Selection of multiple choices for dissemination methods in Pakistan
